# Airway Microbiome in Children with Cystic Fibrosis: A Review of Microbial Shifts and Therapeutic Impacts

**DOI:** 10.3390/medicina61091605

**Published:** 2025-09-05

**Authors:** Georgiana Buruiană, Cristina Mihaela Sima, Dana-Teodora Anton-Păduraru, Aida Corina Bădescu, Cătălina Luncă, Alexandru Duhaniuc, Olivia Simona Dorneanu

**Affiliations:** 1 Department of Preventive Medicine and Interdisciplinarity-Microbiology, “Grigore T. Popa” University of Medicine and Pharmacy, 700115 Iași, Romania; georgiana.buruiana@umfiasi.ro (G.B.); cristina.sima@umfiasi.ro (C.M.S.); alexandru.duhaniuc@umfiasi.ro (A.D.); 2 Clinical Hospital of Infectious Diseases “Sf. Parascheva”, 700116 Iași, Romania; 3 Department of Mother and Child Medicine, “Grigore T. Popa” University of Medicine and Pharmacy, 700115 Iași, Romania; dana.anton@umfiasi.ro; 4 ”Sf. Maria” Children Emergency Hospital, 700309 Iași, Romania; 5 Iasi Regional Center for Public Health, National Institute of Public Health, 700465 Iași, Romania

**Keywords:** cystic fibrosis, pediatric, lung microbiome, CFTR modulators, microbial succession, airway pathogens, antibiotics, probiotics

## Abstract

Even with significant advances in therapeutic interventions and monitoring protocols, cystic fibrosis (CF) remains a critical pediatric health challenge affecting respiratory function and long-term patient outcomes. CF, caused by mutations in the *CFTR* gene, disrupts normal mucociliary clearance and creates conditions for chronic respiratory infections. The disorder affects individuals globally, with pediatric patients facing particularly complex microbial challenges that evolve throughout childhood growth. CF poses significant risks with progressive lung function decline and increased mortality, leading to potential short- and long-term respiratory complications. There is a growing concern among clinicians about the dynamic nature of airway microbial communities, with classical pathogens like *Pseudomonas aeruginosa* and *Staphylococcus aureus* showing sequential emergence patterns that complicate treatment strategies, highlighting an urgent need for microbiome-informed therapeutic approaches. Our review aims to provide a comprehensive overview of airway microbiome evolution in pediatric CF patients. We outline the molecular and ecological mechanisms involved in microbial community progression, as well as the age-related trajectories leading to pathogen-dominated ecosystems and the subsequent complications associated with microbial dysbiosis. Given the widespread implications of disrupted microbial balance on disease progression, our review also presents the temporal landscape of airway microbiome changes, including age-related microbial succession patterns, and explores the underlying mechanisms driving these ecological shifts. The progressive nature of microbial simplification frequently leads to treatment challenges, emphasizing the importance of investigating microbiome-targeted therapeutic interventions. Therefore, in this review, we also explore established therapeutic strategies, including CFTR modulators and probiotics, which could offer promising approaches to maintaining microbial balance and improving outcomes in pediatric CF patients.

## 1. Introduction

Cystic fibrosis is one of the most prevalent autosomal recessive genetic disorders in the United States and among individuals of Caucasian European descent [1]. While traditionally associated with this demographic, recent research highlights its identification, though with generally lower prevalence, in regions such as the Middle East, Asia, and Latin America [2]. The incidence of CF in Europe varies significantly between countries. The highest rate is recorded in Ireland (1:1353), while the lowest rate is reported in Finland (1:25,000), where there is no national newborn screening program, which could contribute to underdiagnosis [3].

CF is a monogenic, chronic, and multisystemic condition resulting from pathogenic mutations in the *CFTR* (cystic fibrosis transmembrane conductance regulator) gene, located on the long arm of chromosome 7 (7q31) [4]. This gene is primarily found in the lungs and pancreas, but also in the sweat glands, salivary glands, intestine, liver, and reproductive tract [4,5]. The CFTR protein functions as a membrane chloride channel, allowing the passage of both chloride and water into mucus-producing cells, thereby reducing the viscosity of secretions. Dysfunction of the CFTR protein disrupts this transport, leading to reduced water content and consequently increased viscosity of secretions across various systems and organs. The presence of mucus secretions with higher viscosity than normal makes their elimination more difficult, leading to obstruction of excretory pathways, as well as the development of inflammatory reactions and infections in the obstructed areas [6,7].

Among all affected systems, the respiratory system experiences the most severe and life-limiting complications [8]. The accumulation of mucus in the lungs promotes microbial colonization and chronic lung inflammation. This persistent inflammatory response contributes to progressive decline in lung function, airflow limitation, bronchiectasis, and ultimately, lung failure [8,9]. Pulmonary involvement in CF begins early in life [9,10,11]. Evidence indicates that CF-related lung disease is often present from infancy, with pulmonary inflammation occurring even before the onset of clinical symptoms [10]. By three months of age, many infants with CF exhibit abnormalities on chest computed tomography (CT), although the relative contributions of inflammation, structural airway changes, and other early pathological processes remain incompletely understood [12,13]. Notably, by the age of three, bronchiectasis has been identified in nearly one-third of children with CF [12]. Understanding these early disease mechanisms may offer opportunities to intervene before irreversible lung damage occurs, with significant implications for clinical management and long-term outcomes.

Historically, therapeutic efforts have focused on a limited number of well-known pathogens, often referred to as the “usual suspects”, identified by culture-based methods, including *Staphylococcus aureus*, *Haemophilus influenzae*, *Pseudomonas aeruginosa*, *Stenotrophomonas maltophilia*, and *Burkholderia cepacia* complex [9,10,14,15]. However, in recent years, culture-independent methods such as 16S rRNA sequencing have revealed that the lungs of patients with CF harbor a far more complex and polymicrobial community than previously recognized [15,16]. A variety of “emerging species” including anaerobes like *Prevotella*, *Veillonella*, *Fusobacterium*, and *Granulicatella*, have been consistently detected in both children and adults with CF, raising questions about their role in disease progression [10]. These species, once dismissed as contaminants or upper airway flora, are now believed to influence airway inflammation, microbial interactions, and potentially modulate the pathogenicity of dominant bacteria such as *P. aeruginosa* [15,16]. For instance, a higher abundance of *Firmicutes* and *Bacteroidetes* correlates with better lung function, whereas a shift toward dominance by Proteobacteria such as *Pseudomonas* and away from commensal anaerobes correlates with declining lung function. The precise clinical significance of these emerging genera remains under investigation, but accumulating evidence suggests that they may either mitigate or exacerbate airway inflammation and thereby influence long-term outcomes in CF lung disease [16,17].

This article presents a narrative literature review designed to comprehensively synthesize current knowledge on the airway microbiome in children with cystic fibrosis, with a particular focus on age-related microbial succession, pathogenic shifts, and therapeutic influences. The search strategy employed a systematic combination of key terms such as “cystic fibrosis,” “lung”, and “microbiota” utilizing Boolean operators (AND, OR) to optimize result specificity and sensitivity. This initial database search yielded 831 articles, which underwent preliminary screening based on title and abstract review to eliminate clearly irrelevant publications.

Following the initial screening phase, full-text articles of potentially eligible studies were obtained and subjected to detailed evaluation. During this comprehensive assessment, additional articles were excluded according to predetermined criteria, including lack of relevance to the research objectives, methodological limitations or incompatibility, misalignment with the study scope, insufficient quality or credibility, language restrictions, and absence of pediatric patient data. Additional relevant studies were identified through manual examination of reference lists from included articles to ensure comprehensive coverage of the literature. In total, 133 studies met all inclusion criteria and were incorporated into this review. Data extraction focused on age-related microbiota composition, the temporal emergence of major pathogens, the role of anaerobic bacteria, the influence of antibiotics and CFTR modulators, comparisons with healthy individuals, and microbial interactions. Extracted findings were synthesized into five thematic domains: (1) the healthy pediatric lung microbiome, (2) microbial succession in cystic fibrosis, (3) key pathogens and their interactions, (4) the role of anaerobes, and (5) the impact of therapeutic interventions. Figures and tables were incorporated to illustrate chronological and ecological patterns, and the integrated analysis was used to identify research gaps and highlight opportunities for early-life microbiome-informed interventions.

## 2. The Healthy Pediatric Lung Microbiome

For decades, the lower respiratory tract was considered a sterile environment, protected from microbial colonization by robust anatomical and immunological defense mechanisms, including mucociliary clearance, alveolar macrophages, and antimicrobial peptides [18]. This long-held dogma suggested that the presence of bacteria in the lungs was indicative of infection or pathological processes [18,19]. However, the advent of culture-independent molecular techniques, particularly high-throughput 16S rRNA gene sequencing, has fundamentally challenged this sterility paradigm and revolutionized our understanding of pulmonary microbiology [19].

The paradigm shift began in 2010 when Hilty and colleagues published one of the first comprehensive culture-independent study of the healthy human lung microbiome, demonstrating the presence of diverse bacterial communities in the airways of both healthy individuals and those with respiratory diseases [20]. This groundbreaking work revealed that the lower respiratory tract harbors a complex, dynamic microbial ecosystem characterized by low biomass and high turnover, with metabolically active bacterial communities that exhibit distinct compositional patterns [21]. Subsequent research has confirmed that the lung microbiome in healthy individuals is primarily derived from microaspiration of upper respiratory tract flora, balanced by continuous clearance mechanisms that maintain the transient and mobile nature of these microbial communities [22].

Current understanding recognizes that a healthy lung microbiome is dominated by taxa such as *Prevotella*, *Streptococcus*, *Veillonella*, and *Actinomyces*, with bacterial loads typically ranging from 10^2^ to 10^3^ colony-forming units (CFU) per mL of bronchoalveolar lavage (BAL) fluid [19]. The resident microbiome plays a crucial role in immune system development, pathogen resistance, and maintenance of respiratory homeostasis, fundamentally altering our approach to understanding both pulmonary health and disease pathogenesis [23].

### 2.1. In Utero Controversy and Initial Colonization

Fetal lungs, like the fetal intestines, were traditionally presumed to be sterile, and infant lungs were thought to acquire their microbial communities exclusively after birth. However, the presence of microbial DNA in placental tissue, amniotic fluid, and fetal meconium has fueled the hypothesis that microbial exposure may begin in utero. While some studies suggest a low-abundance prenatal microbiota [24], others argue these findings are likely artifacts of contamination or low-level translocation of microbial products rather than evidence of viable colonization [25]. Therefore, current consensus suggests that, if present, the prenatal lung microbiome is minimal and transient, with substantive colonization occurring postnatally [25].

### 2.2. Neonatal Period

Immediately following birth, infant mucosal surfaces are quickly populated by microbes derived from the mother’s microbiota. This initial seeding of the neonate’s airways is facilitated by delivery-related exposures and upper airway aspiration. Vaginal delivery is associated with the early presence of genera such as *Lactobacillus*, *Prevotella*, and *Bacteroides*, whereas cesarean section is more likely to result in colonization with *Staphylococcus*, *Corynebacterium*, and other skin-associated taxa [26]. This initial microbial exposure lays the foundation for subsequent community development.

In preterm infants, lung colonization may occur under more dysregulated conditions, including exposure to hospital flora, invasive ventilation, and early antibiotics. In these cases, an aberrant microbiota dominated by *Staphylococcus*, *Ureaplasma*, or *Enterobacteriaceae* may emerge, potentially increasing the risk of bronchopulmonary dysplasia (BPD) or later respiratory morbidity [27].

### 2.3. Infancy (0–12 Months)

In the early months of life, the healthy lung microbiota remains characterized by low biomass (approximately 10^2^–10^3^ CFU per mL of BAL fluid), low evenness, and high turnover. This low biomass is maintained by robust host defenses such as continuous mucociliary clearance, cough reflex, antimicrobial peptides, and efficient immune surveillance, which prevent extensive microbial proliferation and deep colonization. Dominant genera in this dynamic phase include *Streptococcus*, *Veillonella*, *Prevotella*, and *Actinomyces* [19,28]. These organisms likely originate from the upper airways and oral cavity via microaspiration and are transiently present due to continuous mucociliary clearance [19].

Despite its ephemeral nature, this community plays a critical role in immune education. Colonization during this period coincides with a window of heightened immune plasticity, when microbial antigens and metabolites, such as short-chain fatty acids, promote the development of tolerogenic pathways, including regulatory T-cell induction, pulmonary dendritic cell maturation, and balanced Th1/Th2/Th17 responses [23,26].

Factors such as breastfeeding, antibiotic exposure, and environmental microbiota, (siblings, pets, rural living) significantly influence the composition and stability of the developing respiratory microbiota, including the prevalence of *Veillonella* and *Prevotella*, genera linked to immunomodulatory and anti-inflammatory effects in the airways [29,30,31]. In contrast, early antibiotic exposure is known to disrupt microbial colonization patterns and reduce community diversity, potentially affecting immune development and respiratory outcomes [32,33].

### 2.4. Toddler to Preschool Age (1–5 Years)

As children age, their lung microbiota becomes more compositionally stable, reflecting increased microbial exposure and maturation of host-microbe interactions. By age 3–5, the airway microbiota increasingly resembles that of adults, with predominant genera including *Streptococcus*, *Veillonella*, *Prevotella*, *Neisseria*, and *Rothia* [19,30]. While individual variability persists, the shared, core taxa become more consistent.

This period is marked by the emergence of a commensal-dominated core microbiome and a decline in inter-individual variability [19,23]. Notably, genera such as *Provetella* and *Veillonella* appear to persistently dominate the oropharyngeal-lung axis, and their depletion has been associated with asthma, recurrent wheeze, and increased susceptibility to viral infections [30].

Interestingly, even in the absence of overt infection, the lung microbiome remains immunologically active. Resident microbes stimulate epithelial pattern recognition receptors (PRRs), contributing to basal expression of cytokines and antimicrobial peptides that prime the mucosa for pathogen defense without eliciting inflammation [23].

### 2.5. After Age 5

Beyond the age of five, the lung microbiota in healthy children undergoes further stabilization, marked by reduced variability and increasing similarity to adult airway communities. Predominant genera such as *Streptococcus*, *Veillonella*, *Prevotella*, *Neisseria*, and *Rothia* become more consistently represented, forming a reproducible core across individuals [19,31].

This phase reflects not just taxonomic maturation but also functional refinement. Commensal microbes maintain airway immune tone through low-level stimulation of epithelial PRRs, promoting antimicrobial peptide expression and basal cytokine release without inducing inflammation [22,23]. This balanced interaction supports mucosal defense and helps prevent exaggerated immune responses.

Disruption of this stable community, via antibiotics, recurrent infections, or environmental shifts, may interfere with immune regulation and has been linked to increased risk of asthma and wheezing [25,30].

By late childhood, the lung microbiota largely reflects adult composition and function, with implications for immune resilience and long-term respiratory health [19,23,31].

In healthy children, lung colonization begins perinatally, with microbial community assembly proceeding through well-defined developmental phases. Early life is marked by dynamic, low-biomass communities dominated by *Streptococcus*, *Veillonella*, *Actinomyces*, and *Prevotella*, shaped by environmental exposures and host factors. Over time, this community matures into a resilient commensal ecosystem that supports immune homeostasis and respiratory health.

**Figure 1:**
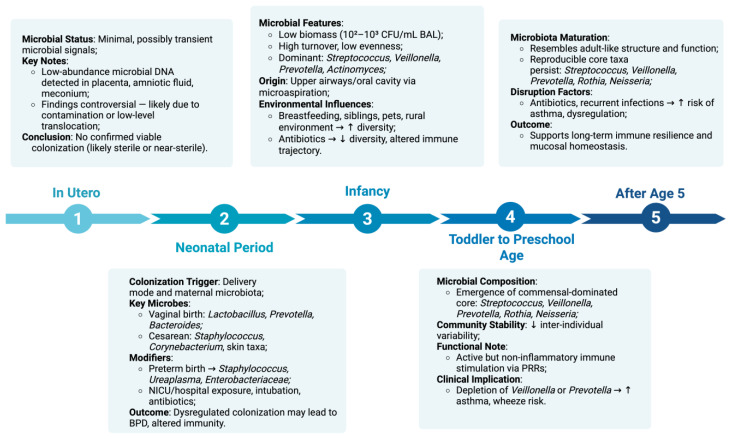
Developmental trajectory of the healthy pediatric lung microbiota across early life stages. The figure illustrates the key phases of lung microbiota acquisition from in utero through late childhood. In healthy children, microbial colonization begins perinatally and proceeds through sequential, age-defined phases: (1) In utero, where microbial DNA detection remains controversial and likely reflects a near-sterile environment; (2) Neonatal period, during which initial seeding is shaped by delivery mode and maternal microbiota; (3) Infancy, marked by low-biomass, high-turnover communities dominated by oral commensals; (4) Toddler to Preschool age, when a stable, commensal-dominated core microbiome emerges; and (5) After age 5, where microbiota maturity is reached, closely resembling adult communities and contributing to mucosal immune homeostasis. Environmental exposures (e.g., antibiotics, breastfeeding, rural living) and clinical factors (e.g., preterm birth, NICU stay) may influence colonization patterns and long-term respiratory health. Image created with BioRender.com. NICU = neonatal intensive care unit.

A refined understanding of this trajectory is essential for identifying early-life microbial disruptions, or dysbiosis, that may predispose to chronic respiratory disease, and for contextualizing deviations observed in conditions like CF or bronchopulmonary dysplasia (Figure 1).

## 3. Microbial Composition and Successional Shifts in the Pediatric CF Lung Microbiome

The lung microbiota in children with CF undergoes a distinct and dynamic evolution from early life. While some studies suggest that the airway microbiota of infants with CF initially resembles that of healthy individuals, dominated by commensals such as *Streptococcus*, *Veillonella*, and *Prevotella*, this similarity is often short-lived [11,34]. As airway inflammation, impaired mucociliary clearance, and antibiotic exposure accumulate, the microbial community shifts toward pathogen dominance and reduced diversity [14,35].

Over time, opportunistic bacteria such as *Staphylococcus aureus*, *Haemophilus influenzae*, and *Pseudomonas aeruginosa* become increasingly prominent, often accompanied by anaerobic and biofilm-forming organisms [15,36]. These changes are closely linked to disease progression and therapeutic response. The following sections explore the succession of microbial communities in pediatric CF, the role of key pathogens and anaerobes, and the influence of antibiotics and CFTR modulators on the airway ecosystem.

### 3.1. Early Colonization Patterns in Cystic Fibrosis

Early life represents a critical window for microbial acquisition in the respiratory tract. In both healthy and CF infants, the initial colonizers are typically oral commensals such as *Streptococcus*, *Veillonella*, and *Prevotella*, which are believed to reach the lungs through microaspiration of upper airway secretions [11,16]. These early colonizers help shape the microbial ecosystem and may influence immune tolerance and airway development.

While some studies report that the airway microbiota of infants with CF resembles that of healthy subjects during the first months of life, other findings suggest that divergence can occur early, with reduced diversity and altered community structure already evident within the first year [37]. These differences may reflect the influence of the *CFTR* mutation on mucus viscosity and mucociliary clearance, creating a niche that gradually selects for pathogenic taxa [14,35,38].

Several perinatal and environmental factors also influence early colonization patterns. Mode of delivery, breastfeeding, antibiotic exposure, and the household microbial environment all contribute to the initial seeding and development of the lung microbiota [39]. Infants delivered via vaginal birth are typically colonized by maternal vaginal and fecal microbes, such as *Lactobacillus* and *Prevotella*, while those born by cesarean section often exhibit delayed microbial acquisition, lower diversity, and enrichment in skin- or hospital-associated taxa like *Staphylococcus* [39,40]. Breastfeeding promotes colonization by beneficial bacteria such as *Bifidobacterium* and *Lactobacillus*, and provides immunological factors that help shape microbial composition and protect against pathogen overgrowth [39]. In contrast, early antibiotic exposure, common in CF care, has been associated with reduced microbial richness and accelerated emergence of pathogenic organisms in both the gut and airways [14,41].

Although originally described in healthy infants, these early-life factors play an equally influential role in shaping the respiratory microbiome in CF, where they interact with the underlying genetic and physiological disturbances, they are also highly relevant to CF [16,39]. In children with CF, these early-life factors interact with an already altered airway environment, characterized by impaired clearance and inflammation, potentially amplifying dysbiosis and accelerating microbial succession toward pathogen-dominated communities [14,16,39]. Because early microbial communities may influence future pathogen acquisition and airway inflammation, interventions during this window could offer critical opportunities to alter disease trajectory in CF [16].

### 3.2. Microbial Succession and Age-Related Shifts in Children with Cystic Fibrosis

The airway microbiota in children with CF evolves in distinct, age-related stages, reflecting the combined effects of impaired mucociliary clearance, chronic inflammation, and repeated antibiotic exposure or other treatments [35,38,41]. Unlike healthy children, who typically acquire greater microbial diversity with age, CF patients show early microbial divergence, progressive ecological simplification, and a shift toward domination by a limited set of pathogenic species [10,14,42]. These changes are strongly associated with declining lung function, increased exacerbation risk, and disease progression [43,44].

#### 3.2.1. Infancy (0–12 Months)

The first year of life represents a crucial period for airway microbial development in children with CF [10,11]. In the first months, the CF airway microbiota shows high diversity and is typically dominated by oral-associated genera such as *Streptococcus*, *Veillonella*, *Prevotella*, and *Rothia* [11,45]. At this stage, the bacterial load is generally low, and airway inflammation is minimal [11]. The microbiota composition may still closely resemble that of healthy controls, particularly in asymptomatic infants.

However, even during these early time points, some CF infants, particularly those with respiratory symptoms, may exhibit early microbial divergence characterized by increased *Staphylococcus* abundance and declining *Streptococcus* populations [11]. This early shift suggests that CF-related alterations in microbial composition can begin within the first months of life, potentially initiating the inflammatory-microbial feedback loop earlier than previously recognized.

By 3 months of age, many CF infants without early respiratory symptoms continue to maintain a microbiota composition similar to healthy controls, with sustained dominance by oral commensals [10,11]. However, symptomatic infants increasingly demonstrate microbial divergence, with notable shifts toward opportunistic pathogens.

The impact of clinical management practices becomes apparent during this period. Ahmed et al. (2019) demonstrated that *Streptococcus* species remained dominant throughout infancy in a CF cohort receiving continuous antistaphylococcal prophylaxis, while *S. aureus* was rarely detected [15]. These findings contrast with cohorts not receiving prophylaxis, highlighting how early antibiotic interventions can significantly influence microbial succession patterns [11].

As infancy progresses, microbial diversity may begin to decline in some patients, especially those exposed to frequent antibiotics or experiencing early airway inflammation [37,43]. Opportunistic pathogens such as *S. aureus*, *H. influenzae*, and *Moraxella catarrhalis* become increasingly prevalent in certain populations [45].

Early anaerobic colonizers, particularly *Veillonella* and *Prevotella*, appear frequently in infant CF airways and have been proposed to support microbial diversity and potentially delay pathogen expansion [11,39]. These organisms may form protective polymicrobial communities that compete with pathogenic species for resources and colonization sites. However, current evidence for their protective role remains mixed and largely correlative, requiring further investigation to establish causal relationships [11,39,44].

The presence and persistence of these anaerobes during infancy may represent an important window for intervention, as maintaining microbial diversity during this critical period could influence long-term disease trajectories and delay the typical progression toward pathogen-dominated communities.

#### 3.2.2. Toddlerhood (1–3 Years)

During toddlerhood, microbial succession becomes more apparent. The relative abundance of commensal genera continues to decline, while intermittent colonization by *P. aeruginosa* begins to emerge in some patients. *S. aureus* often remains one of the dominant organisms during this stage, while *P. aeruginosa* may first appear in low abundance but gradually establish itself, particularly in inflamed and mucus-obstructed airways [15,42].

Anaerobic genera such as *Prevotella*, *Veillonella*, and *Fusobacterium* have been detected in the lower airways of young children with CF, including toddlers, and may persist despite antibiotic therapy [44,46]. These bacteria may form part of polymicrobial communities that influence the behavior of co-infecting pathogens [46]. Although many anaerobes are associated with healthy oral environments, their persistence under hypoxic conditions in the CF lung could contribute to inflammation or support the virulence of dominant pathogens like *P. aeruginosa* [47].

Goddard et al. demonstrated that bacterial communities in the lower airways differed substantially from those of the upper airways in young children with CF, highlighting the importance of direct lower airway sampling for accurate microbial characterization in this population [43].

#### 3.2.3. Preschool to Early Childhood (3–6 Years)

As children age into the preschool years, the airway microbiota becomes increasingly dominated by classic CF pathogens. *P. aeruginosa*, *Achromobacter xylosoxidans*, and *Stenotrophomonas maltophilia* are detected with greater frequency [10,35]. *S. aureus* frequently remains a dominant organism, while *H. influenzae* can still be detected in a subset of patients. Commensals become less abundant, and overall microbial diversity declines [10].

Anaerobic genera such as *Prevottella*, *Veillonella*, *Fusobacterium* and *Peptostreptococcus* have been detected in the lower airway of young children with CF, including those in the preschool age range.

#### 3.2.4. Late Childhood to Adolescence (7–18 Years)

By late childhood and adolescence, many patients exhibit a low-diversity, pathogen-dominated airway microbiota. *P. aeruginosa* often becomes the dominant species, forming persistent biofilms and developing significant antimicrobial resistance. *S. aureus*, including methicillin-resistant strains (MRSA), frequently co-colonizes [48]. Other pathogens commonly detected during this stage include *Burkholderia cepacia* complex, *S. maltophilia*, *A. xylosoxidans*, and *Mycobacterium abscessus* [35,41].

Notably, *M. abscessus*, a rapidly growing non-tuberculous mycobacterium (NTM), emerges in some older pediatric CF patients, particularly those with structural lung damage or advanced disease [35]. Though less prevalent than classical CF pathogens, its clinical significance is substantial. *M. abscessus* is intrinsically resistant to many antibiotics, forms biofilms, and can persist intracellularly, making it especially difficult to eradicate [35,48]. Its presence is associated with worsened lung function and the need for prolonged, multi-drug antibiotic therapy [41].

In advanced disease stages, the persistent presence of pathogenic microbes such as *P. aeruginosa* and *M. abscessus* drives chronic neutrophilic inflammation, leading to tissue damage and airway remodeling. This cyclical interaction between infection and immune response creates a self-perpetuating inflammatory environment that further disrupts microbial balance and accelerates lung function decline [14,44].

**Figure 2:**
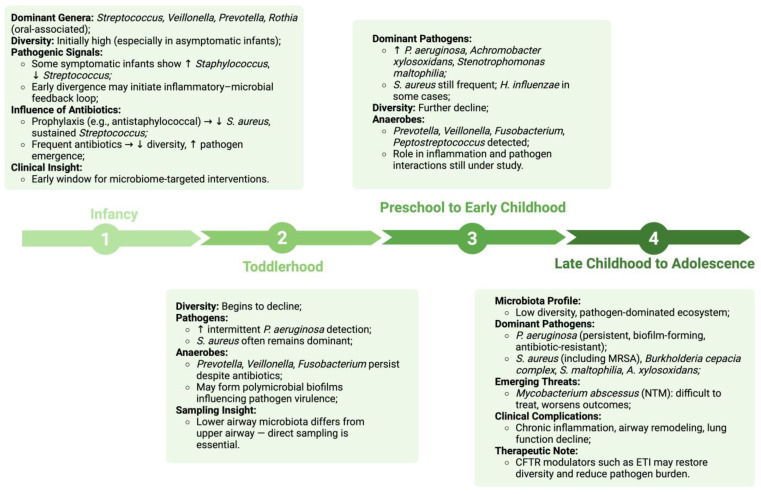
Microbial succession in pediatric cystic fibrosis airways. This figure illustrates the age-specific progression of airway microbial communities in children with cystic fibrosis, from infancy through adolescence. Early life is characterized by diverse communities dominated by oral commensals such as *Streptococcus*, *Veillonella*, and *Prevotella*. Over time, microbial diversity declines, and classical CF pathogens, including *Pseudomonas aeruginosa*, *Staphylococcus aureus*, and the *Burkholderia cepacia* Complex, become increasingly dominant. Anaerobic bacteria persist throughout and may modulate inflammation or interact with co-colonizing pathogens. Clinical and therapeutic implications specific to each developmental stage are also indicated. Image created with BioRender.com.

Emerging data suggest that CFTR modulator therapies, such as elexacaftor–tezacaftor–ivacaftor, may partially restore microbial diversity and reduce pathogen load, potentially interrupting the typical trajectory of microbial succession in CF airways [35,49]. Microbial succession in pediatric CF is characterized by early-life diversity followed by progressive ecological simplification and pathogen dominance. This trajectory varies between individuals but typically begins within the first months of life and intensifies with age and disease severity. Mapping these age-specific changes helps identify windows of opportunity for early intervention and supports the development of microbiome-informed treatment strategies (Figure 2).

### 3.3. Pathogenic Microbes in Pediatric CF Lungs

As the airway microbiota in children with CF matures, a distinct group of pathogenic bacteria begins to dominate the microbial landscape. These organisms are acquired in a relatively consistent sequence from infancy through adolescence, shaped by the altered mucus environment, impaired mucociliary clearance, chronic inflammation, and frequent antibiotic exposure [10,11]. Unlike in healthy children, where microbial diversity tends to increase with age, the CF airway undergoes progressive ecological simplification, favoring a small number of opportunistic and often antibiotic-resistant taxa [14,42].

This shift toward pathogen dominance has important clinical consequences: infections with organisms such as *S. aureus*, *H. influenzae*, *P. aeruginosa*, and *Burkholderia cepacia* Complex are associated with increased airway inflammation, lung function decline, and reduced treatment responsiveness [15,43]. Moreover, newer pathogens such as *A. xylosoxidans* and *S. maltophilia* are increasingly detected in pediatric cohorts, often coexisting with dominant species and contributing to persistent infection [41,47].

This chapter reviews the major bacterial pathogens implicated in pediatric CF, highlighting their typical timing of appearance, clinical relevance, and potential interactions with other microbial or host factors (Table 1).

#### 3.3.1. *Staphylococcus aureus*

In children with CF, *S. aureus* is among the first and most common bacteria to establish airway colonization. It is routinely identified through culture and sequencing methods as early as infancy [11,14,69]. This organism often appears prior to *P. aeruginosa* and can persist as the dominant species during the preschool period. Studies monitoring pediatric CF populations have found that over 90% of patients are colonized with *S. aureus* by the age of three [70]. Such chronic presence has been associated with heightened airway inflammation, diminished microbiota diversity, and increased use of antimicrobials [20,71].

The rise of methicillin-resistant *S. aureus* (MRSA) presents serious challenges in CF care, owing to its β-lactam resistance and mechanisms supporting persistence. Between 2001 and 2014, the proportion of CF patients in the United States with MRSA raise from 2% to approximately 26.5%. MRSA colonization has been strongly associated with worsened clinical outcomes, including accelerated loss of lung function, a higher frequency of exacerbations, and nearly double the risk of death or need for lung transplant [70].

In contrast to the United States, MRSA remains less prevalent in European pediatric CF cohorts. Data comparing national registries reveal that *S. aureus* infections, both methicillin-sensitive and methicillin-resistant, are notably more common among U.S. children with CF than in the UK. These disparities likely stem from differing clinical practices, including infection control strategies, antibiotic use, and early eradication approaches. The analysis also highlighted that U.S. patients receive more aggressive early treatments, such as higher rates of rhDNase (recombinant human deoxyribonuclease) and hypertonic saline use, which may influence pathogen colonization dynamics and associated outcomes [72].

Among MRSA strains, the USA300 clone has emerged as particularly concerning. This community-acquired lineage is increasingly detected in pediatric CF populations [51,70]. It carries potent virulence elements such as Panton–Valentine leukocidin (PVL), which contributes to cell damage, and the arginine catabolic mobile element (ACME), which promotes epithelial adherence and enhances bacterial fitness [15,51]. These attributes enable USA300 to thrive in the inflamed CF lung and evade immune clearance.

*S. aureus* adapts to the CF airway by acquiring mutations in regulatory genes such as *agr* (accessory gene regulator), *sarA* (staphylococcal accessory regulator), and *sigB (*alternative sigma factor B), which collectively modulate toxin production, surface adhesion, and stress responses [50,51]. These mutations result in decreased expression of cytotoxic factors and enhanced biofilm formation, promoting a phenotype better suited for persistence in the hostile CF airway environment [15]. Additionally, the emergence of thymidine-dependent small-colony variants, characterized by impaired cell separation, reduced hemolysin production, and long-term intracellular survival, further supports the transition toward chronic infection rather than acute virulence [73]. The adapted form is more resistant to immune mechanisms and better suited to survive in the CF lung.

Biofilm formation is a critical factor in this persistence, especially in the CF environment, where acidic pH, neutrophil-derived oxidants, and stagnant mucus offer a protective niche for adherent bacterial communities [74]. These biofilms impair both immune responses and antibiotic access, making eradication more difficult.

One distinctive adaptation of *S. aureus* in CF is the development of small colony variants (SCVs). These are slow-growing, metabolically altered subtypes with diminished pigmentation and hemolysis, increased tolerance to antibiotics, particularly aminoglycosides, and a strong capacity to form biofilms [50]. SCVs emerge in response to oxidative stress and prolonged exposure to antimicrobial agents [15,51], and they have the ability to persist within host epithelial cells, thereby avoiding immune recognition and drug activity.

Although traditionally considered a dominant CF pathogen, *P. aeruginosa* does not always displace *S. aureus*. In fact, coinfection is common and may persist over time, especially in pediatric cases [75]. Furthermore, *P. aeruginosa* may promote the selection of *S. aureus* SCVs, which are better equipped to withstand both bacterial competition and oxidative stress [76].

The presence of *Staphylococcus aureus*, particularly MRSA, has been associated with adverse clinical outcomes in early life, including lower weight-for-height percentiles, poorer nutritional status, and a greater likelihood of progression from CFTR-related metabolic syndrome (CRMS) to a confirmed diagnosis of CF. These effects appear most prominent in infancy and early childhood, a critical period when microbial colonization and inflammation may shape long-term lung health [77].

While early eradication strategies are employed in many countries, there is no universally accepted regimen. Protocols including rifampicin may offer temporary bacterial clearance, but robust evidence for lasting clinical benefit remains limited [78]. Treatment approaches vary between centers, and further pediatric-specific clinical trials are essential to establish best practices.

#### 3.3.2. *Pseudomonas aeruginosa*

*Pseudomonas aeruginosa* is a hallmark pathogen in CF, typically emerging after the age of two and progressively dominating the pediatric airway with advancing disease. Even early, intermittent colonization has been associated with increased airway inflammation and significantly poorer lung function outcomes by age five, underscoring its impact during critical periods of pulmonary development [11,52].

Persistence of *P. aeruginosa* in the CF airway is supported by multiple adaptive mechanisms. One of the most prominent is biofilm development, which confers protection against both host immune defenses and antibiotic treatment [79,80]. Within these biofilms, the bacterium often undergoes phenotypic changes, including the transition to mucoid variants characterized by excessive alginate production-features strongly associated with chronic infection and accelerated clinical decline [41,81].

*P. aeruginosa* exhibits significant metabolic flexibility and virulence. It produces a wide array of enzymes (elastase, alkaline protease, catalase) and redox-active metabolites like phenazines that contribute to epithelial damage and oxidative stress [81,82]. These secreted factors exacerbate neutrophilic inflammation, even when the pathogen is present at low abundance [52].

Its presence reshapes the airway microbiota. Over time, *P. aeruginosa* displaces anaerobic commensals such as *Veillonella* and *Prevotella*, driving a transition from diverse microbial communities to low-complexity profiles dominated by a few pathogens [83,84]. Network analyses reveal antagonistic interactions between *P. aeruginosa* and these commensals, which may limit recovery of microbial diversity once *P. aeruginosa* becomes established [82].

Polymicrobial interactions further complicate the role of *P. aeruginosa* in CF. Coinfection with *S. aureus* is common in early childhood and is associated with an increased risk of pulmonary exacerbations and accelerated lung function decline [11,15]. *P. aeruginosa* can also induce *S. aureus* to shift into SCVs, a phenotype associated with increased antibiotic tolerance and oxidative stress resistance [51,76].

Environmental and treatment pressures, including frequent antibiotic courses and the formation of oxygen-depleted niches in the mucus-rich CF lung, create selective conditions favoring the dominance of *P. aeruginosa* [16,35]. Prophylactic or targeted regimens against other microbes, such as antistaphylococcal strategies or long-term macrolide therapy used to control NTM, can unintentionally disrupt the microbial balance, reducing beneficial anaerobic or commensal species and providing ecological space for *P. aeruginosa* to expand [3,13,54]. Over time, this contributes to reduced diversity and increased airway inflammation, which further selects for organisms that thrive in hostile, inflamed environments [14]. These pressures, compounded by biofilm formation and phenotypic adaptation of *P. aeruginosa*, make eradication increasingly difficult as infection becomes chronic.

The pathogen is detected in up to 90% of CF patients using molecular methods, and its abundance correlates with advancing age and disease severity [36,53]. Histopathological studies demonstrate that *P. aeruginosa* contributes to bronchial wall thickening, alveolar damage, and neutrophilic infiltration, hallmarks of progressive CF lung disease [85].

Although early eradication protocols and the introduction of CFTR modulators such as elexacaftor—tezacaftor—ivacaftor (ETI) have contributed to a decline in *P. aeruginosa* detection rates, long-term persistence of this pathogen remains a significant concern. Evidence suggests that despite reductions in bacterial load, clonal *P. aeruginosa* strains can persist for over 20 months following modulator initiation, often maintaining mucoid phenotypes and antibiotic resistance profiles. These findings highlight the limitations of modulator therapy in completely eliminating chronic infections and reinforce the need for targeted antimicrobial strategies against *P. aeruginosa* as a continuing priority in CF management [3,86].

#### 3.3.3. *Haemophilus influenzae*

*Haemophilus influenzae* is a key early colonizer of the CF airway and one of the most frequently detected pathogens in infants and young children with CF. Its prevalence is significantly higher in CF infants than in healthy controls, with detection in up to 40% of BAL fluid samples during early life, underscoring its clinical relevance in disease onset and progression [11]. Early colonization is frequently associated with increased neutrophilic inflammation and a decline in clinical status [69].

The pathogen’s persistence in the lower airways is supported by its ability to form biofilms, which confer resistance to both host immune mechanisms and antimicrobial treatments. These biofilms have been documented in clinical isolates from children with CF and are associated with chronic inflammation and poor microbial clearance [56,87]. Furthermore, biofilm-associated *H. influenzae* strains show altered expression of adhesins and a capacity for intracellular survival, contributing to long-term persistence in the airway [55,88].

Molecular studies have also demonstrated significant antimicrobial resistance among *H. influenzae* strains isolated from pediatric CF patients. Roberts et al. reported macrolide resistance in numerous isolates, with resistance genes linked to prolonged antibiotic exposure [89]. Genomic analyses further reveal substantial strain diversity and resistance determinants, even among closely related isolates [90], suggesting rapid adaptation within the CF lung environment.

Experimental models using synthetic CF sputum media have shown that *H. influenzae* can thrive under nutrient-limited, anaerobic conditions mimicking the CF airway, thereby enhancing its survival and resistance potential [91]. These conditions may also promote interactions with other microbes, including early colonizers like *S. aureus*, which together can exacerbate inflammation and accelerate airway damage [16,84].

Comparative studies have shown that *H. influenzae* is frequently detected in CF infants prior to age two, sometimes persisting intermittently over time [16]. While some strains are transient, even short-term colonization has been linked to increased cytokine levels and microbial community shifts that reduce diversity and promote pathogenic dominance [50].

The interplay between *H. influenzae* and other organisms, such as *Streptococcus pneumoniae*, may influence its persistence. For instance, competitive exclusion in settings in vitro has shown that *S. pneumoniae* can suppress *H. influenzae* growth under certain conditions, potentially affecting early microbial succession in CF lungs [11].

In conclusion, *H. influenzae* plays a critical role in early CF lung disease. Its ability to persist via biofilm formation, develop antimicrobial resistance, and interact with other microbes highlights its importance as a therapeutic target in young CF patients. Continuous monitoring and targeted interventions are essential to reduce its long-term impact on airway health.

#### 3.3.4. *Stenotrophomonas maltophilia*

*Stenotrophomonas maltophilia* has emerged as a relevant multidrug-resistant opportunistic pathogen in the lungs of children with CF. It is commonly isolated alongside other aerobic bacteria such as *S. aureus* and *H. influenzae*, particularly in conventional culture-based studies of CF sputum [35]. While *S. maltophilia* was traditionally considered a late colonizer, recent studies suggest that it is increasingly detected in pediatric populations due to enhanced microbiological surveillance and longer survival of CF patients [60].

Evidence from epidemiological studies confirms its presence in both children and adolescents, particularly those with more advanced lung disease, previous colonization by *P. aeruginosa*, and frequent antibiotic exposure [61]. The median age of first detection is often during adolescence, but colonization in younger children is becoming more common. Risk factors contributing to early *S. maltophilia* emergence include broad-spectrum antibiotic therapy, corticosteroid use, and coinfection with *Aspergillus fumigatus* [60].

Although once considered a passive colonizer, *S. maltophilia* is now linked to clinically significant outcomes, such as accelerated FEV1 decline, increased hospitalization, and greater need for lung transplantation [60]. These associations are particularly evident during chronic infection phases. Moreover, *S. maltophilia* infections frequently co-occur with other pathogens, amplifying the risk for exacerbation [61].

The pathogen’s intrinsic and acquired resistance mechanisms, including efflux pumps and antibiotic-modifying enzymes, limit treatment options [60]. Standard antibiotics such as trimethoprim-sulfamethoxazole, minocycline, and levofloxacin retain partial efficacy, but no universally accepted eradication protocol exists. Over-treatment poses a risk of selecting further resistant strains [92].

Genomic studies reveal a high degree of strain heterogeneity, suggesting adaptive evolution within the CF airway. This diversity may affect antibiotic susceptibility and virulence, although its impact on clinical outcomes remains underexplored [60,92].

Taken together, *S. maltophilia* represents a growing challenge in pediatric CF care. Improved surveillance, resistance profiling, and targeted antimicrobial strategies are urgently needed to reduce its long-term impact on lung health.

#### 3.3.5. *Burkholderia cepacia* Complex

The *Burkholderia cepacia* Complex (BCC) refers to a group of genetically distinct but phenotypically similar Gram-negative bacteria that pose a significant clinical concern in CF care. These organisms are highly adaptable and intrinsically resistant to many antibiotics, making them particularly difficult to treat [58,93]. Although less frequently isolated than classic CF pathogens such as *P. aeruginosa* or *S. aureus*, BCC species are strongly associated with worsened clinical outcomes in children and adolescents with CF [50,94,95].

Aerobic bacteria commonly detected in CF sputum include *S. aureus*, *H. influenzae*, *P. aeruginosa*, *S. maltophilia*, and BCC. Their presence typically correlates with advanced stages of lung disease [36,94].

At least 24 species have been identified within the complex, with the most clinically relevant in CF including: *B. cenocepacia*, *B. multivorans*, *B. vietnamiensis*, *B. dolosa*, and *B. cepacia* [58]. Among these, *B. cenocepacia*, particularly the ET-12 epidemic clone, is regarded as the most virulent. It is associated with *cepacia syndrome*, a fulminant and often fatal necrotizing pneumonia and sepsis, and significantly poorer outcomes, including rapid lung function decline and high post-transplant mortality [59,96].

Children infected with BCC, especially with ET-12 strains, experience more frequent pulmonary exacerbations even when baseline FEV1 is preserved [96]. These findings underscore the aggressive nature of certain BCC strains and their disproportionate burden in pediatric populations.

In many centers, the presence of BCC, particularly *B. cenocepacia*, is considered a contraindication for lung transplantation due to poor post-transplant survival [59,97].

BCC’s ability to evade immune clearance is further enhanced by its interaction with the host immune system. Chronically infected children exhibit altered immune profiles, including reduced IL-17F levels and impaired glucose metabolism, which may exacerbate inflammation and reduce the host’s ability to clear infection. These immune changes, along with persistent colonization, contribute to significantly lower survival rates in BCC-infected children compared to their non-infected cohort members [57].

Molecular and epidemiological studies have shown a shift in species prevalence over time. For instance, a Canadian study revealed that *B. multivorans* has become more prevalent than *B. cenocepacia*, possibly due to strict infection control protocols [95]. However, *B. multivorans* still carries clinical risk, although generally less severe than *B. cenocepacia* [96].

BCC strains exhibit remarkable resistance to multiple drug classes due to various mechanisms, including efflux pumps, β-lactamase production, and decreased outer membrane permeability [93]. This multidrug resistance has earned BCC a place on global priority pathogen lists [58]. Treatment regimens are often ineffective, and no standard eradication protocol exists.

Within the airway microbiome of children with CF, members of the BCC tend to predominate in environments characterized by low microbial diversity, particularly in those with chronic colonization by *P. aeruginosa* [34]. These *Burkholderia*-dominated communities often coexist with other opportunistic pathogens such as *S. aureus* and *Achromobacter* spp., forming polymicrobial groups that have been associated with heightened airway inflammation and more severe clinical trajectories [98].

New therapies, including bacteriophage treatment, are under investigation, but face significant barriers such as strain-specificity, difficulty penetrating CF mucus, and regulatory uncertainty [97]. Nevertheless, they offer a potential path forward in treating otherwise refractory infections.

Taken together, the BCC presents a multifaceted and serious health threat in pediatric CF, characterized by high virulence, antibiotic resistance, immune evasion, and poor clinical outcomes. Ongoing species-level surveillance, stringent infection control, and development of novel therapeutic options remain critical in managing these challenging infections.

#### 3.3.6. *Achromobacter* spp

*Achromobacter* spp., particularly *Achromobacter xylosoxidans*, have become increasingly detected in the airways of children with CF, with prevalence rates in the United States estimated between 3% and 8% [62,99]. Although once considered a low-virulence organism, recent studies have demonstrated that *A. xylosoxidans* infection is associated with more severe disease in pediatric CF patients, including lower lung function, more frequent exacerbations, and faster clinical decline [64,100,101].

Marsac et al. performed a multicenter, retrospective case–control study and observed that children who acquired *A. xylosoxidans* had significantly reduced lung function at the time of infection compared to matched controls. Over the subsequent two years, these patients experienced increased rates of pulmonary exacerbations, required more frequent hospitalizations, and had higher usage of both intravenous and oral antibiotics [100]. In a similar vein, Bar-On et al. reported that individuals with either intermittent or chronic *Achromobacter* infections exhibited a more rapid decline in FEV_1_ and more frequent exacerbations than those with only transient bacterial detection [64].

In children with CF, those who acquired *Achromobacter* spp. showed a markedly faster annual reduction in lung function, experiencing a decline of 9.07% per year, compared to just 1.18% in their uninfected counterparts [101].

The clinical management of *Achromobacter* infections in CF is particularly challenging due to the organism’s intrinsic resistance to multiple antibiotic classes, including aminoglycosides, tetracyclines, and β-lactams. In a compassionate use program, cefiderocol, a novel siderophore cephalosporin, was administered to eight CF patients, two of whom were children, suffering from extensively drug-resistant *A. xylosoxidans*. While clinical improvement was observed in most cases, microbiological relapse occurred in 11 of the 12 courses administered, highlighting the pathogen’s capacity to persist despite targeted therapy [63].

Other members of the genus, such as *Achromobacter ruhlandii*, have also been isolated from pediatric CF patients. In one case, plasmid profiling from *A. ruhlandii* strains obtained over time from the same child revealed consistent plasmid content, including resistance genes and toxin–antitoxin systems, suggesting a role in long-term colonization and microbial adaptability [102].

The use of CFTR modulators like ETI appears to influence the composition of the airway microbiota. A decreased prevalence of *A. xylosoxidans* has been observed among children receiving ETI compared to previous cohorts, suggesting a possible impact of this therapy on infection patterns [99].

Collectively, these findings highlight the clinical significance of *Achromobacter* spp. in pediatric CF, emphasizing its association with disease progression, antimicrobial resistance, and the need for targeted therapeutic approaches.

#### 3.3.7. *Mycobacterium abscessus*

*Mycobacterium abscessus* (Mabs) is recognized as one of the most clinically significant NTM in CF. It is considered the most virulent NTM, combining multiple intrinsic and acquired resistance mechanisms, and is strongly associated with accelerated pulmonary decline and frequent treatment failure [65].

Taxonomically, *M. abscessus* is a rapidly growing mycobacterium that comprises three subspecies: *M. abscessus* subsp. *abscessus*, *M. abscessus* subsp. *massiliense*, and *M. abscessus* subsp. *bolletii*. While *M. abscessus* and *M. massiliense* are most frequently encountered and have been linked to dominant circulating clones (DCCs) across CF populations worldwide, *M. abscessus* subsp. *bolletii* is reported less often, but remains clinically important due to similar resistance mechanisms and pathogenic potential [68,103,104]. A highly similar clone of *M. abscessus* subsp. *massiliense* has even been detected in geographically distinct CF and non-CF outbreaks spanning three continents, suggesting wide adaptability and potential transmissibility [103].

Epidemiological data show that NTM infection is uncommon in young children but becomes increasingly prevalent during adolescence and remains frequent in adulthood. Within this distribution, *M. abscessus* tends to appear earlier in life than *M. avium* complex (MAC), with affected patients usually younger and demonstrating poorer lung function. While MAC predominates in the United States, *M. abscessus* is responsible for the majority of CF-associated NTM infections in Europe and Australia, accounting for 50–80% of cases [65]. Infections are a major source of morbidity and mortality in CF, and have been suspected to involve person-to-person transmission [68]. Studies in pediatric CF centers have documented clusters of nearly identical isolates differing by fewer than seven single nucleotide polymorphisms, supporting cross-infection events [67]. However, genomic similarity alone cannot conclusively prove direct transmission [68].

Management of *M. abscessus* remains extremely challenging. Treatment regimens are long, multidrug-based, and often poorly tolerated, typically combining a macrolide with intravenous amikacin and one or more companion agents such as imipenem, cefoxitin, or tigecycline [65]. Even with such intensive protocols, outcomes are frequently suboptimal due to high levels of intrinsic and acquired resistance. Biofilm formation within the CF airway and the ability to adopt a non-replicating “persister” state in oxygen- and nutrient-limited microenvironments further promote persistence, while advanced structural lung damage restricts antibiotic penetration [66].

Because of these limitations, novel therapeutic strategies are being explored. One innovative approach is experimental clinical application of bacteriophage therapy, which offers targeted activity against drug-resistant strains. In a reported case, intravenous administration of two engineered mycobacteriophages together with antibiotics produced rapid evidence of bacterial lysis, radiological improvement by three months, and negative airway cultures by four months. Remarkably, the explanted lungs after transplantation showed no detectable *M. abscessus*, illustrating the potential of phage therapy as a complementary tool when conventional antibiotics fail [66].

### 3.4. Anaerobes

Anaerobic bacteria are increasingly recognized as common and functionally relevant members of the CF lung microbiota, including in pediatric patients. Studies have reported their presence in high abundance and diversity across all age groups, often being detected even in early disease stages. In a comprehensive culture- and sequencing-based study, 91.1% of CF sputa were positive for at least one anaerobic species, identifying 31 genera and 69 distinct anaerobic species, the broadest diversity ever documented in CF lungs [46]. Common genera include *Prevotella*, *Veillonella*, *Fusobacterium*, *Atopobium*, *Peptostreptococcus* and *Porphyromonas* [36,46]. These bacteria are often part of the healthy oral microbiota and may enter the lungs via microaspiration, persisting even under antibiotic pressure [105].

Anaerobes appear to play a complex role in pediatric CF lung health and disease progression. Some studies link a higher abundance of *Veillonella* and other anaerobes with better lung function (FEV1 > 70%) and slower decline in respiratory health [17,46]. Notably, *Porphyromonas catoniae* has emerged as a potentially protective member of this group. In a pediatric cohort, Keravec et al. found that *P. catoniae* was significantly more abundant in CF patients who remained uninfected by *P. aeruginosa* [106]. Importantly, its decline preceded *P. aeruginosa* acquisition, suggesting its potential as an early biomarker for infection risk [106].

However, other anaerobes may contribute to inflammation. *Prevotella melaninogenica*, *Veillonella parvula* and *Fusobacterium nucleatum* produce short-chain fatty acids (SCFAs), such as acetate and butyrate, as fermentation by-products. These SCFAs are detected in CF sputum and BAL and activate the host’s G-protein coupled receptor 41 (GPR41) on airway epithelial cells. This signaling cascade upregulates IL-8 secretion, a potent neutrophil chemoattractant, thereby directly linking microbial metabolism with neutrophilic inflammation [105,107].

Interactions between anaerobes and classical CF pathogens add further complexity. In vitro studies showed that *V. parvula* formed biofilms together with *P. aeruginosa*, resulting in greater biomass compared to mono-species biofilms [105]. Similarly, high co-occurrence rates between anaerobes and *P. aeruginosa* have been observed in CF sputum, suggesting persistent ecological association [36]. Anaerobes contribute structurally and metabolically to polymicrobial biofilms, shielding pathogens from antibiotics and immune responses. Fermentative anaerobes also release metabolic products such as lactate, acetate, and succinate, which can serve as substrates for aerobes like *P. aeruginosa* under hypoxic conditions, promoting interspecies metabolic cross-feeding and ecological stability [105]. These cooperative behaviors likely enhance resilience and antibiotic tolerance, especially within mixed-species biofilms [47].

Some anaerobes also contribute to antibiotic resistance at a community level. *Prevotella* spp. isolated from CF patients have been shown to produce β-lactamases, which degrade β-lactam antibiotics. This confers indirect protection to neighboring pathogens, a process known as “passive resistance” [105]. As a result, even non-pathogenic anaerobes can enhance the resilience of more virulent bacteria like *P. aeruginosa* and *S. aureus* within shared microbial communities.

Emerging hypotheses suggest that anaerobes may also influence colonization by NTM, either by modulating host immunity or altering microbial competition. Although direct evidence is limited, this ecological interaction remains a topic of interest, particularly in patients with polymicrobial dysbiosis [105].

Despite their prevalence, the clinical significance of targeting anaerobes during exacerbations remains debated. A retrospective study assessing outcomes of exacerbations treated with antibiotics active against anaerobes (clindamycin, metronidazole) found no consistent clinical benefit, suggesting that therapeutic targeting may need to be personalized depending on community structure and host status. Some patients with anaerobe-dominated profiles may still derive benefit [108]. Carmody et al. emphasized that microbial shifts during exacerbations are heterogeneous, some patients experience notable changes in anaerobic populations, while others remain compositionally stable, highlighting the need for individualized, microbiome-informed treatment strategies [109].

Modern molecular methods such as extended culture, qPCR, and shotgun metagenomics have been instrumental in detecting and characterizing anaerobes, which are often missed by standard culture. Even under chronic antibiotic pressure, obligate anaerobes persist in the CF lung ecosystem [46,110]. Their contribution to inflammation, microbial stability, or dysbiosis likely depends on the specific species involved and their interactions with other community members.

## 4. Influence of Therapeutic Interventions on the Pediatric Cystic Fibrosis Lung Microbiome

CF is a complex genetic disease marked by chronic respiratory infections, inflammation, and malnutrition due to CFTR dysfunction [111]. Standard care includes frequent antibiotic use to control pulmonary infections, but this may disrupt microbial balance and select for resistant organisms, particularly in early childhood [112].

In parallel, the introduction of CFTR modulators, especially the triple combination ETI, has significantly improved clinical outcomes in pediatric patients by enhancing mucociliary clearance and reducing pathogen burden [111,113]. Moreover, microbiome-targeted interventions such as probiotics are gaining interest for their potential to modulate systemic inflammation and support gut–lung axis signaling, contributing to better respiratory and nutritional status [114,115].

This chapter will explore the interplay between antibiotics, CFTR modulators, and probiotics in shaping the microbiome and clinical trajectory in children with CF.

### 4.1. CFTR Modulators and Their Influence on Lung Microbiome and Infection Patterns

The F508del mutation, the most prevalent CFTR defect in CF, results in the synthesis of a misfolded CFTR protein. This misfolded form is recognized and retained within the endoplasmic reticulum (ER)**,** where it is prematurely degraded, preventing its transport to the epithelial cell surface [116]. Consequently, very little CFTR reaches the apical membrane, and the small amount that does is often unstable and exhibits reduced channel opening and gating efficiency [117]. These molecular defects significantly impair chloride and bicarbonate transport across epithelial surfaces, which contributes to dehydrated airway secretions, increased mucus viscosity, and ineffective mucociliary clearance. This dysfunction fosters an environment conducive to chronic bacterial colonization and ongoing inflammation in the lungs [118].

CFTR modulators are therapeutic agents designed to enhance the function of the defective CFTR protein by targeting different stages of its processing and function. These compounds are classified into several types:

- Potentiators—such as ivacaftor, which enhance the gating function of CFTR channels already present at the cell surface, allowing chloride ions to flow more effectively.
- Correctors—including lumacaftor, tezacaftor, and elexacaftor, which assist in proper protein folding and facilitate trafficking of CFTR to the apical membrane.
- Amplifiers–like nesolicaftor, currently in experimental stages, which aim to increase the overall production of CFTR protein by boosting gene expression [118].
- Read-through agents—exemplified by ataluren (PTC124), are developed for Class I CFTR mutations caused by premature stop codons. These agents facilitate the ribosome’s ability to bypass the stop signal, enabling synthesis of a full-length CFTR protein. Laboratory studies have demonstrated partial recovery of channel activity, yet large clinical trials have shown inconsistent clinical benefits, with modest improvements in chloride transport but no sustained gains in lung function or exacerbation reduction. Current research is exploring their use in combination with other modulator classes to enhance therapeutic outcomes [119,120,121].
- Stabilizers—experimental compounds designed to increase the persistence of CFTR protein at the apical cell surface, reducing its premature internalization and degradation. This approach is particularly suited for mutations where functional CFTR reaches the membrane but exhibits reduced stability. Although none have received regulatory approval, preclinical evidence indicates that combining stabilizers with correctors and potentiators may prolong CFTR activity and improve long-term channel function [119,120,121].

The combination therapy consisting of ETI currently represents the most effective treatment available for individuals with at least one F508del CFTR mutation. It has been approved in the United States, Europe, and other high-income countries. However, access remains restricted in many low- and middle-income regions due to high treatment costs and regulatory challenges [118,122].

Beyond their established role in correcting ion transport and enhancing pulmonary function, CFTR modulators, particularly the triple-combination therapy ETI, have also been associated with significant alterations in the airway microbiota [117]. Extended-culture and molecular analyses have shown that ETI use reduces both the prevalence and abundance of classical CF pathogens, with these effects being especially notable in pediatric populations [99,123]. For instance, *P. aeruginosa* colonization has been reported to decline following ETI initiation [117,124]. Additionally, *A. xylosoxidans* detection was lower in children receiving ETI compared to earlier, pre-modulator cohorts [99]. López Cárdenes et al. further observed a general reduction in microbial burden in pediatric CF patients treated with CFTR modulators. These findings suggest a broader reshaping of the airway microbial ecosystem in response to improved epithelial function [125].

These reductions may reflect enhanced mucociliary clearance, reduced mucus viscosity, decreased airway inflammation, and overall altered surface conditions that are unfavorable for these pathogens [117,118].

CFTR modulator therapy in pediatric patients with CF has been consistently associated with significant alterations in the respiratory microbiota. Notably, treatment with ivacaftor and combination regimens such as lumacaftor/ivacaftor or ETI leads to a marked decline in the prevalence and abundance of classical CF pathogens, including *P. aeruginosa*, *S. aureus*, and *H. influenzae* [86,122,123,126]. These changes reflect a partial reversal of the pathogen-dominated dysbiosis commonly observed in the CF airway. Simultaneously, modulator therapy may permit the persistence or even enrichment of anaerobic genera such as *Prevotella*, *Veillonella*, and *Fusobacterium*, which were previously suppressed in pathogen-dense communities [86,123]. These taxa may occupy ecological niches vacated by dominant pathogens and are thought to contribute to a more diverse but potentially inflammation-modulating microbial environment.

Several studies report a post-treatment increase in alpha diversity, suggesting a trend toward microbiome re-equilibration and potential restoration of airway microbial homeostasis [117,123]. In children aged 6 to 11, early initiation of modulators has been particularly effective in reducing pathogenic colonization and promoting a shift toward a more balanced microbial community [122,126].

In summary, CFTR modulators, particularly ETI, have revolutionized CF care by improving CFTR protein function, enhancing ion transport, and altering the biochemical landscape of the airway. These changes correlate with reduced colonization by key pathogens but may also lead to the persistence of anaerobic or low-abundance bacteria, the clinical relevance of which is still being defined. Continued surveillance of the lung microbiome and better understanding of post-modulator microbial dynamics will be essential for optimizing antimicrobial strategies in this new therapeutic era.

### 4.2. Antibiotic Interventions and Their Influence on the Pediatric Cystic Fibrosis Lung Microbiome

Antibiotics remain essential for managing pulmonary infections in children with CF. Intravenous therapy is commonly employed during acute exacerbations, with agents such as ceftazidime, tobramycin, and piperacillin/tazobactam being most frequently used [127]. Additional intravenous options like colistin, meropenem, and ciprofloxacin are typically reserved for multidrug-resistant organisms, particularly *P. aeruginosa* [128]. For chronic suppressive therapy, inhaled antibiotics such as tobramycin and aztreonam are standard [127]. Given the prevalence of *P. aeruginosa* respiratory infections in the pediatric CF population, inhaled tobramycin solution constitutes the most frequently prescribed antimicrobial agent in the management of these patients [129]. Despite its therapeutic efficacy, aminoglycoside-related nephrotoxicity represents a substantial clinical concern, as evidenced by the 22% incidence of acute kidney injury during aminoglycoside treatment courses in pediatric CF patients [130].

In early childhood, prophylactic antibiotic regimens are frequently employed to prevent *S. aureus* colonization, though practices vary by country. For instance, CF centers in Australia routinely administer amoxicillin-clavulanate during infancy, a strategy not adopted in the United States [131]. In the United Kingdom, long-term use of azithromycin is licensed from six months of age [132]. These differing approaches reflect the ongoing debate regarding the benefits and risks of prophylaxis. While some studies suggest that early *S. aureus* suppression may improve outcomes, others caution that prophylactic antibiotics can reduce microbial diversity and potentially favor the emergence of non-traditional or pathogenic taxa [131].

Importantly, a comparison of BAL samples from 32 infants with CF on prophylaxis versus those not receiving prophylaxis, conducted in cohorts from Australia and the United States, found no major differences in the relative abundance of most genera, except for *Fusobacterium* spp., which was significantly more abundant in those not on prophylaxis [15]. This suggests a specific sensitivity of *Fusobacterium* to early antibiotic exposure, underscoring the nuanced effects of prophylaxis on the developing lung microbiome.

While antibiotics are essential for infection control, they impose selective pressure on the microbial ecosystem. In infants with CF, prophylactic and therapeutic antibiotics have been consistently linked to reduced microbial diversity [9,131]. In some cases, *Streptococcus* becomes dominant following antibiotic use, a shift associated with poorer pulmonary outcomes [131]. Moreover, longitudinal analyses indicate that antibiotic exposure, rather than age alone, is a primary driver of microbial diversity decline in early life [133].

Beta-lactams, especially with prolonged use, are associated with the emergence of multidrug-resistant Gram-negative pathogens like *Burkholderia* and *Stenotrophomonas* [134]. These regimens can also shift the microbiota composition toward *Proteobacteria*, while reducing anaerobic taxa [131]. Despite compositional changes, total bacterial load often remains stable, indicating the persistence or recovery of a resilient microbial core [133].

Polymicrobial infections, including combinations of *P. aeruginosa*, *S. aureus*, *H. influenzae*, anaerobes, and *S. maltophilia*, are frequently treated with broad-spectrum empirical regimens [135]. However, these approaches may fail to eliminate non-dominant or co-infecting species, especially anaerobes, thus enabling microbial shifts through reduced competition [133]. Notably, some anaerobes, like *Prevotella* spp., produce β-lactamases that can inactivate antibiotics, shielding pathogens like *P. aeruginosa* via “passive resistance” [105].

This ecological dynamic contributes to the persistence of dominant pathogens and complicates eradication. Repeated courses of antipseudomonal therapy also drive the evolution of multidrug-resistant *P. aeruginosa*, further narrowing treatment options. Despite frequent regimen changes during hospitalization, sustained clinical improvement is not always achieved, highlighting the need for microbiome-informed strategies [136].

Altered pharmacokinetics in CF makes antibiotic dosing a challenge. Since CF patients have increased clearance rates for many common antibiotics, including β-lactams, aminoglycosides, fluoroquinolones, and trimethoprim, they often require higher or more frequent doses compared to non-CF populations [137]. Due to a notable lack of robust evidence to guide optimal intravenous regimens, as highlighted by Cochrane reviews, individualized therapy is crucial [138]. For this reason, Therapeutic Drug Monitoring (TDM) is especially vital for aminoglycosides to effectively balance therapeutic benefit with the risk of nephrotoxicity. Moreover, nebulizer system design can significantly impact drug delivery, serum concentration, and patient adherence, further reinforcing the need for personalized treatment plans [139].

Azithromycin, in addition to antimicrobial effects, exerts anti-inflammatory and immunomodulatory functions, reducing exacerbation frequency and dampening airway inflammation even in *P. aeruginosa*-negative patients. It also inhibits *P. aeruginosa* virulence and biofilm formation via quorum sensing disruption [132].

Despite extensive antibiotic exposure, the CF lung microbiome often demonstrates ecological resilience, with dominant taxa re-emerging over time. However, transient shifts induced by antibiotics are linked to fluctuations in inflammation and lung function [140]. Notably, antibiotic prophylaxis has been associated with reduced airway neutrophils and IL-8, suggesting potential anti-inflammatory benefits [9]. These trade-offs underscore the need for longitudinal studies evaluating the long-term consequences of early-life microbial modulation.

#### Antibiotic Treatment and Extra-Intestinal Consequences

Antibiotics are fundamental in the management of pulmonary exacerbations in children with CF, but their repeated and prolonged use has important consequences for the host microbiota. The STOP2 randomized trial demonstrated that 10 days of intravenous antibiotics was non-inferior to 14 days for early responders, and that 21 days was not superior to 14 days for poor responders, suggesting that treatment length can be individualized without loss of efficacy [141]. Despite routine intravenous use, systematic reviews highlight a lack of high-quality evidence to guide the optimal duration of therapy, and at least 25% of patients fail to recover baseline lung function following exacerbation treatment [138]. Beyond clinical outcomes, antibiotic exposure profoundly shapes microbial communities. In the gut, children with CF exhibit a marked reduction in microbial richness and diversity from early childhood, and these alterations are strongly linked to repeated antibiotic courses [142]. Dysbiosis in the gastrointestinal tract is further associated with functional impairment; increased abundance of *Escherichia coli* correlates with malabsorption and intestinal inflammation [143]. Importantly, the impact of antibiotic-induced dysbiosis extends beyond the gut. Upper airway studies in infants with CF show that nasal microbiota is perturbed following respiratory tract infections and antibiotic exposure, even before chronic colonization becomes established [144].

Dysbiosis also influences susceptibility to non-respiratory infections. A large pediatric cohort identified 108 urinary tract infection (UTI) episodes among 826 CF patients, with unique risk factors including CF-related diabetes, distal intestinal obstruction syndrome, and nephrolithiasis. Unlike in the general population, UTIs in CF were less often due to Gram-negative rods and more frequently caused by *Enterococcus faecalis* [145]. This altered uropathogen profile likely reflects systemic consequences of chronic antibiotic exposure and host microbial imbalance.

Taken together, these findings highlight that antibiotic therapy in pediatric CF not only requires balancing efficacy and toxicity, but also carries long-term risks of gut, airway, and urinary microbiome disruption. Recognition of these broader effects underscores the need for individualized treatment duration, microbiome-sparing approaches, and exploration of adjunct therapies such as probiotics or symbiotics.

### 4.3. Probiotics and Their Potential Role in Pediatric Cystic Fibrosis Management

The use of probiotics in CF has garnered increasing interest as a supportive therapeutic approach, particularly in pediatric populations where microbial dysbiosis and chronic inflammation are prominent from early life. While most probiotic interventions have traditionally targeted gastrointestinal symptoms, emerging evidence suggests potential benefits that extend to respiratory health through systemic immune modulation and the gut—lung axis [112,146,147].

Two randomized controlled trials have reported a reduction in pulmonary exacerbation rates in children with CF receiving probiotic supplementation. Bruzzese et al. demonstrated that daily administration of *Lactobacillus rhamnosus GG* (LGG) significantly decreased the frequency of pulmonary exacerbations and hospitalization rates in pediatric patients [146]. LGG refers to a well-characterized strain of *L. rhamnosus* (ATCC 53103), originally isolated by Sherwood Gorbach and Barry Goldin, hence the initials “GG”. This strain has been extensively studied and is considered one of the most well-researched probiotics, noted for its acid and bile resistance, strong adhesion to intestinal mucosa, and immunomodulatory and antimicrobial effects, including the secretion of bacteriocins and modulation of epithelial responses [148]. Similarly, Rahmani et al. found that *Lactobacillus reuteri* supplementation led to improvements in FEV1 and a lower rate of exacerbations in children under five years old [149]. These findings suggest a broader clinical benefit that may extend beyond pulmonary inflammation.

Probiotics may exert their effects by modulating systemic inflammation. A meta-analysis by Cruz Mosquera et al. highlighted that strains such as *Lactobacillus reuteri* and LGG were associated with reductions in inflammatory markers, including IL-6, IL-8, and fecal calprotectin [147]. However, their impact on pulmonary function tests and exacerbation frequency was variable across studies.

In the study by Ray et al., oral supplementation with *Lactobacillus* resulted in a significant increase in gut *Bifidobacteria*, which was associated with improved weight gain and reduced pulmonary exacerbation rates in children with CF. The authors suggested that these clinical improvements were likely mediated by modulation of intestinal microbiota, which in turn influenced systemic immune responses, providing support for the involvement of the gut–lung axis in CF pathophysiology [112].

Although most probiotic studies in CF target the gastrointestinal tract, indirect effects on the lung microbiota have been hypothesized via the gut–lung axis. Probiotic-induced reductions in systemic inflammation may create a less permissive environment for pathogens such as *P. aeruginosa* or *H. influenzae* [112,148]. While direct evidence for lung microbiota modification remains limited, improved airway outcomes, such as fewer exacerbations and stabilized lung function, observed in clinical studies support their inclusion as adjunctive therapies [146,149].

Regarding prebiotics and symbiotic, the available clinical evidence in CF remains limited and inconclusive. Prebiotics have rarely been investigated, and current data do not support their routine use. Clinical trials assessing symbiotic indicate that they are safe and well tolerated but have not shown consistent benefits in terms of lung function, exacerbation rates, or quality of life. At present, both prebiotics and symbiotic should be considered experimental approaches, with larger and longer-term randomized trials needed to clarify their true role in pediatric care [147].

Probiotics may offer a non-invasive, low-risk strategy to support clinical stability in pediatric CF, particularly in younger patients with heightened inflammation and microbial imbalance. Current data support their role in reducing exacerbation rates and modulating immune responses, though the mechanistic links to lung microbiota composition require further study. Future trials should aim to define strain-specific effects, optimal dosing regimens, and long-term safety to guide clinical implementation.

## 5. Conclusions and Future Directions

Despite coordinated efforts by healthcare systems aimed at managing and treating it, CF remains a substantial pediatric respiratory challenge. CF presents significant risks due to its progressive nature, high rates of morbidity and mortality, as well as the potential for patients to develop severe short- and long-term pulmonary complications. Clinicians should be mindful of the complex microbial dynamics that influence disease progression in patients experiencing unfavorable clinical courses.

While CF airway microbiome dysfunction is a notable clinical concern, the mechanisms underlying microbial succession and pathogen dominance remain incompletely understood. Enhanced understanding of these processes could offer opportunities for developing novel therapeutic targets. Furthermore, gaining insight into the factors that drive early microbial divergence could support the development of preventive therapeutic strategies, which would be particularly advantageous for pediatric populations at risk of rapid disease progression.

Health authorities are increasingly concerned by the growing complexity of airway infections caused by CF pathogens that exhibit resistance, including multidrug resistance, against common antimicrobials such as beta-lactams, aminoglycosides, and quinolones. The emergence of antimicrobial resistance in CF pathogens is linked to frequent antibiotic exposure and the selective pressure of the altered airway environment. With the increase in antimicrobial resistance rates, there is a pressing need for microbiome-informed therapeutic options.

CFTR modulators have emerged as transformative interventions; however, studies on their long-term effects on microbial communities have revealed varying results across different patient populations. Therefore, there is a growing need for further research to explore the interactions between CFTR restoration and various microbial taxa, assessing their potential benefits in maintaining airway health. Another therapeutic approach involves the strategic use of probiotics and microbiome-targeted interventions, which could serve as valuable tools in reducing inflammation and exacerbation rates. Currently, microbiome-based therapies remain largely experimental in CF care.

Advances in multi-omics approaches are revolutionizing our understanding of CF airway microbiome dynamics. Integrative analyses combining microbiome, metabolome, and proteome data have revealed functional shifts that correlate with disease progression and treatment outcomes, offering promising avenues for personalized medicine. Early-life studies underscore the critical importance of microbial acquisition patterns during infancy as determinants of long-term respiratory health, highlighting the need for targeted interventions during these developmental windows.

Several microbiome-informed strategies are under investigation for maintaining microbial balance in CF, with clinical trials exploring probiotic interventions, personalized antimicrobial approaches, and multi-omics-guided therapeutic protocols. Since the development of precision microbiome medicine appears feasible, greater efforts should be directed towards this goal. This initiative stands to benefit not only children in developed countries with access to advanced therapies but also pediatric patients in resource-limited settings and high-risk populations who may benefit from cost-effective, microbiome-targeted interventions.

## Figures and Tables

**Table 1:** Key pathogens in pediatric cystic fibrosis lung disease.

Pathogen Name	Typical Age of Emergence/Prevalence	Key Virulence Factors/Characteristics	Clinical Significance	
*Staphylococcus aureus*	Early Childhood	Biofilm formation, exotoxins, antibiotic resistance (MRSA)	Early inflammation, exacerbations, common co-infection	[50,51]
*Pseudomonas aeruginosa*	School-Age, Adolescence	Biofilm (mucoid phenotype), alginate, quorum sensing, efflux pumps	Chronic infection, progressive lung damage, exacerbations	[52,53,54]
*Haemophilus influenzae*	Early Childhood	Biofilm, IgA protease	Early colonization, acute exacerbations	[55,56]
*Burkholderia cepacia* Complex	Later Childhood, Adolescence	High intrinsic resistance, biofilm, “cepacia syndrome”	Severe lung decline, poor prognosis, highly transmissible	[57,58,59]
*Stenotrophomonas maltophilia*	Later Childhood, Adolescence (increasing in younger patients)	Intrinsic multidrug resistance, biofilm formation, efflux pumps	Associated with FEV1 decline, increased hospitalizations, coinfections, and poor outcomes	[35,60,61]
*Achromobacter xylosoxidans*	Variable, increasing	High intrinsic resistance, biofilm	Associated with lung decline, exacerbations	[62,63,64]
*Mycobacterium abscessus*	Rare in young children. increasingly prevalent during adolescence	Most virulent NTM, biofilm formation, ability to enter non-replicating “persister” state	Accelerated lung decline, high morbidity/mortality, possible cross-infection in pediatric CF centers	[65,66,67,68]

## Abbreviations

The following abbreviations are used in this manuscript:

CF	Cystic fibrosis
CFTR	Cystic fibrosis transmembrane conductance regulator
CT	Computed tomography
BAL	Bronchoalveolar lavage
BPD	Bronchopulmonary dysplasia
NICU	Neonatal intensive care unit
PRRs	Pattern recognition receptors
NTM	Non-tuberculous mycobacterium
MRSA	Methicillin-resistant *Staphylococcus aureus*
rhDNase	Recombinant human deoxyribonuclease
PLV	Panton-Valentin leucocidin
ACME	Arginine catabolic mobile element
Agr	Accessory gene regulator
SarA	Staphylococcal accessory regulator
SigB	Alternative sigma factor B
SCVs	Small colony variants
CRMS	CFTR—related metabolic syndrome
ETI	Elexacaftor/Tezacaftor/Ivacaftor
BCC	*Burkholderia cepacia* Complex
Mabs	*Mycobacterium abscessus*
DCCs	Dominant circulating clones
MAC	*Mycobacterium avium* complex
SCFAsGPR41	Short-chain fatty acids G-protein coupled receptor 41
ER	Endoplasmatic reticulum
TDM	Therapeutic drug monitoring
UTI	Urinary tract infection
LGG	*Lactobacillus rhamnosus* GG
